# Lipids and lipoproteins may play a role in the neuropathology of Alzheimer’s disease

**DOI:** 10.3389/fnins.2023.1275932

**Published:** 2023-11-16

**Authors:** Omer Akyol, Sumeyya Akyol, Mei-Chuan Chou, Shioulan Chen, Ching-Kuan Liu, Salih Selek, Jair C. Soares, Chu-Huang Chen

**Affiliations:** ^1^Molecular Cardiology, Vascular and Medicinal Research, The Texas Heart Institute, Houston, TX, United States; ^2^NX Prenatal, Houston, TX, United States; ^3^Department of Neurology, Kaohsiung Medical University Hospital, Kaohsiung Medical University, Kaohsiung, Taiwan; ^4^Graduate Institute of Medicine, College of Medicine, Kaohsiung Medical University, Kaohsiung, Taiwan; ^5^Institute of Precision Medicine, College of Medicine, National Sun Yat-sen University, Kaohsiung, Taiwan; ^6^Department of Psychiatry and Behavioral Sciences, UTHealth Houston McGovern Medical School, Houston, TX, United States

**Keywords:** Alzheimer’s disease, lipids, cholesterol, electronegative LDL, LDLR, LOX-1

## Abstract

Alzheimer’s disease (AD) and other classes of dementia are important public health problems with overwhelming social, physical, and financial effects for patients, society, and their families and caregivers. The pathophysiology of AD is poorly understood despite the extensive number of clinical and experimental studies. The brain’s lipid-rich composition is linked to disturbances in lipid homeostasis, often associated with glucose and lipid abnormalities in various neurodegenerative diseases, including AD. Moreover, elevated low-density lipoprotein (LDL) cholesterol levels may be related to a higher probability of AD. Here, we hypothesize that lipids, and electronegative LDL (L5) in particular, may be involved in the pathophysiology of AD. Although changes in cholesterol, triglyceride, LDL, and glucose levels are seen in AD, the cause remains unknown. We believe that L5—the most electronegative subfraction of LDL—may be a crucial factor in understanding the involvement of lipids in AD pathology. LDL and L5 are internalized by cells through different receptors and mechanisms that trigger separate intracellular pathways. One of the receptors involved in L5 internalization, LOX-1, triggers apoptotic pathways. Aging is associated with dysregulation of lipid homeostasis, and it is believed that alterations in lipid metabolism contribute to the pathogenesis of AD. Proposed mechanisms of lipid dysregulation in AD include mitochondrial dysfunction, blood–brain barrier disease, neuronal signaling, inflammation, and oxidative stress, all of which lead ultimately to memory loss through deficiency of synaptic integration. Several lipid species and their receptors have essential functions in AD pathogenesis and may be potential biomarkers.

## 1. Introduction

Neurodegeneration refers to ongoing neuron and synapse loss in specific brain regions, leading to disorders like Alzheimer’s disease (AD), Parkinson’s disease, and Huntington’s disease. AD is characterized by the presence of neurofibrillary tangles and senile plaques in the brain. Key factors in the pathogenesis of AD include β-amyloid protein (Aβ) buildup and neuronal apoptosis. Current therapies cannot fully prevent progression due to permanent neuron loss (Citron, 2010) but can slow it down. A clear-cut relationship between lipids/lipoproteins/apolipoproteins and AD has not yet been established, even though there are signs of abnormalities in lipid metabolism. Despite extensive preclinical and clinical research over the past three decades, no disease-preventive or disease-modifying treatment alternatives have been identified for AD and late-onset clinical dementia.

In this article, we review and characterize the role of lipids and lipoproteins in the neuropathology of AD and have focused on their impact on amyloid precursor protein (APP) processing, tau phosphorylation, and Aβ production. Lipids and lipoproteins play a key role in the processing of APP and the production of Aβ. Abnormal phosphorylation of tau protein, also a hallmark feature of AD, can be affected by lipids and lipoproteins. Therefore, a better understanding of the mechanistic role of these molecules in AD pathology may provide avenues for therapeutic interventions.

## 2. Hypothesis

**Figure 1:**
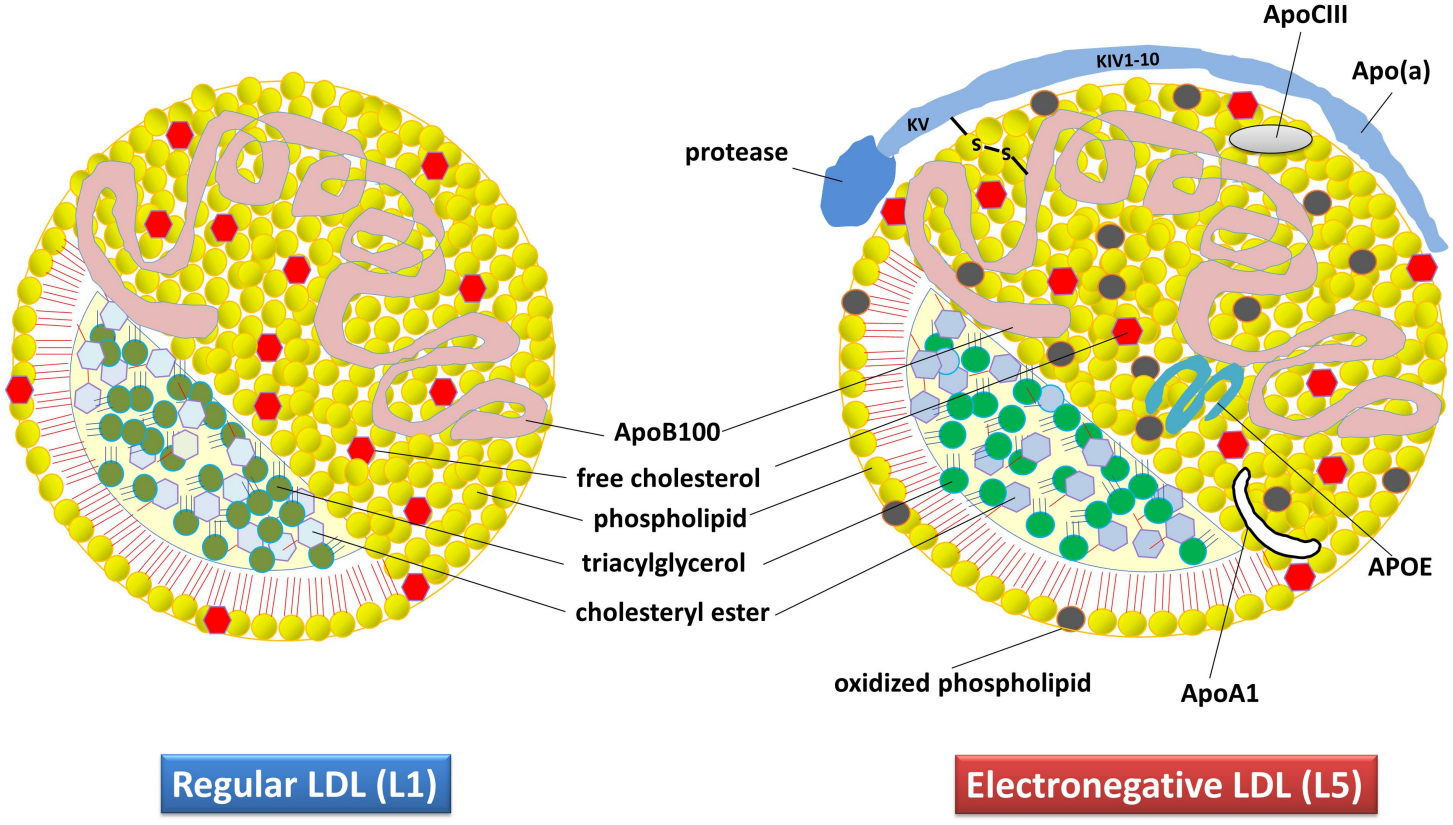
Composition and structural characteristics of regular (L1) and electronegative (L5) LDL in humans. The apolipoprotein content specifies the structure, receptor interactions, assembly, and metabolism of these lipoproteins. The L5 subtype differs from regular LDL because of its additional apolipoproteins. Apo(a), apolipoprotein small (a); ApoA1, apolipoprotein-A1; ApoB100, Apolipoprotein-B100; ApoCIII, apolipoprotein-CIII; APOE, apolipoprotein-E.

The pathophysiology of AD involves multiple genetic, environmental, developmental, social, biochemical, and anatomical factors. The speculative presumption has been that reactive oxygen species (ROS) play a key role in AD by oxidizing lipids, which leads to the modification of low-density lipoprotein (LDL) particles through various mechanisms, primarily oxidation. This modification results in the formation of a subclass of products known as negatively charged LDL. The major modifiable risk factors of AD, such as lifestyle (diet, alcohol, smoking, etc.), traumatic brain injury, type 2 diabetes, and vascular disease (Edwards Iii et al., 2019), are all linked to LDL metabolism and lipoprotein disruption. Our hypothesis is that LDL, especially its most electronegative subfraction called L5 (Figure 1), may play a key role in AD pathophysiology.

Vascular risk factors, such as type 2 diabetes, hyperlipidemia, smoking, obesity, high blood pressure, and physical inactivity, can reduce cerebral perfusion, activate neuroinflammatory responses, and increase ROS production. These risk factors may induce Aβ production and cause intellectual disability in the form of cognitive decline. Elevated plasma LDL cholesterol levels are more likely to be related to early onset AD, and gene-based rare variant testing has shown that rare apoB-coding variants were significantly more abundant in early-onset AD (Wingo et al., 2019).

Human and animal investigations have demonstrated that the pathophysiology of AD is directly related to impaired lipid metabolism. Recent studies have shown that L5 has an important role in the initiation and evolution of atherosclerosis and in the increased interrelationship of vasoconstriction with Aβ. The combined effects of Aβ and ROS may lead to cognitive impairment through cerebrovascular dysfunction. Therefore, both ROS-mediated lipoprotein changes and L5, the major product of these oxidative changes, may be considered as potential biomarkers of metabolic stress and may be important in the pathogenesis of AD and other types of dementia (Akyol et al., 2020).

## 3. Supporting evidence

### 3.1. Contemporary pathophysiology of AD

There is increasing evidence that cerebrovascular disease (CVD) affects the pathogenesis of AD. Co-occurrence of CVD and AD pathology is common in older patients with dementia (Attems and Jellinger, 2014), and vascular pathology images in the brain are regularly monitored in MRI scans of AD patients (Staffaroni et al., 2017). Observational epidemiological investigations have found that lifestyle and cardiovascular risk factors are correlated with dementia risk, and targeting these risk factors may be a feasible prevention strategy for dementia (Norton et al., 2014). In two independent commissioned reports by the *Lancet* and *National Academy of Medicine*, encouraging results suggest that modifying risk factors including hypertension, diabetes, physical inactivity, and obesity could prevent up to 35% of cases of dementia (Livingston et al., 2017; National Academies of Sciences and Medicine, 2017).

In a recent meta-analysis by Bellenguez et al. a two-stage, genome-wide association study led to the validation of 33 previously identified loci. This groundbreaking research not only effectively doubled the overall number of genetic loci linked to AD risk, but also significantly contributed to our knowledge in the field by expanding our understanding of AD’s underlying pathophysiology (Bellenguez et al., 2022). The most prominent gene sets uncovered in this study were closely tied to amyloid/tau, whereas several other gene sets with significant associations pertained to lipid metabolism, endocytosis, and immune processes, including the activation of macrophages and microglial cells. The studies have shown that cardiovascular-related loci elevate the risk of late-onset AD. The ε4 allele of apolipoprotein-E (APOE), a major risk factor for AD, encodes a lipid transporter required in the metabolism of cholesterol (Mahley, 2016). The FINGER study, using 11C-Pittsburgh compound B-PET imaging, found a link between early brain Aβ accumulation and declining executive functions and a strong APOE4 association with Aβ positivity (Kemppainen et al., 2018). Genome-wide association studies have identified a single nucleotide polymorphism (SNP) in late-onset AD that is involved in lipid processing (Lambert et al., 2013; Reitz, 2013) and cholesterol metabolism (Carmona et al., 2018). Apart from APOE, 90 SNPs on 19 chromosomes elevated the risk of both AD and cardiovascular issues. Four novel genetic variants that increase AD risk were identified in a meta-analysis across three independent cohorts (Broce et al., 2019). In AD-affected brains, three genes showed differential expression changes, suggesting shared risk with cardiovascular disease (Broce et al., 2019).

Clinical, epidemiological, and animal studies suggest that elevated plasma lipids (i.e., hypercholesterolemia) are risk factors for AD (Xue-Shan et al., 2016). Neuronal cells are the target for cholesterol binding via LDL receptors (LDLR). Hypercholesterolemia can impair the blood brain barrier (BBB), leading to metabolic disintegration of cholesterol in the brain (Xue-Shan et al., 2016). Collectively, these findings indicate a common genetic relationship between AD and plasma lipids, with particular genes identified as potential drivers of this genetic link.

Individuals with AD may have both vascular and AD pathology, potentially worsening cognitive decline (Broce et al., 2019). Newly discovered genetic loci in MBLAC1, MINK1, and MTCH2/SPI1 may increase susceptibility to brain inflammation and vascular lesions, leading to worse AD outcomes (Broce et al., 2019). Creating a multi-gene “hazard” model for cardiovascular pathways could help predict when older adults might develop AD (Desikan et al., 2017) and allow for targeted therapeutic intervention to alter its course.

#### 3.1.1. Oxidative stress in linking L5 to AD

Several studies have revealed a significant correlation between inflammatory and vascular risk factors and AD, along with a strong association between dementia and factors such as nitrative and oxidative stress (Perry et al., 2002; Fitzpatrick et al., 2004). AD patients have elevated synaptic cholesterol release and neuronal degeneration, potentially contributing to the susceptibility of lipoproteins to ROS, leading to the generation of oxidized LDL (ox-LDL). This ox-LDL could serve as a stable biomarker for monitoring the inflammatory cascade in AD (Kankaanpaa et al., 2009).

A positive correlation between plasma NO concentration and oxidative stress marker 3 nitrotyrosine (3-NT) levels (Sinem et al., 2010) suggests insights into AD’s mechanisms and pathophysiology, potentially linked to ROS-induced stress. When the body is exposed to disease, infection, or certain environmental stressors, oxidation surpasses the level of antioxidants, and huge amounts of highly reactive ROS begin to accumulate (Zhao et al., 2014). AD patients show markers of lipid peroxidative oxidative stress in the hippocampus and cortex. Elevated NO levels in animal studies are associated with Aβ neurotoxicity, potentially contributing to AD development (Luth et al., 2001). Additionally, plasma ox-LDL levels are higher in AD patients than in controls (Zhao et al., 2014), but they negatively correlate with Mini-Mental State Exam (MMSE) scores. These findings may relate to increased cerebral cholesterol concentration and oxidative/nitrative stress. In another study, plasma ox-LDL levels were significantly higher in AD patients than in those with normal cognitive function (Dildar et al., 2010). However, an earlier study found little change in plasma ox-LDL levels in AD patients, particularly in severe cases (Zafrilla et al., 2006).

#### 3.1.2. BBB disruption in AD

Impairment of the BBB is a significant factor in AD pathogenesis (Montagne et al., 2015; van de Haar et al., 2016). The damaged BBB allows proinflammatory and neurotoxic factors, such as thrombin, immunoglobulins, hemoglobin, bacterial breakdown products, iron, and fibrinogen, to disrupt CNS function and promote neuroinflammation and neurodegeneration (Zlokovic, 2011). Recent research suggests that BBB dysfunction is an early event leading to mild cognitive damage in AD (van de Haar et al., 2016), offering a potential target for intervention. However, despite increased understanding, clinical strategies to prevent BBB disruption in AD are still lacking. Vascular risk factors significantly impact the incidence of vascular dementia and AD (Gudala et al., 2013). A meta-analysis revealed a 73% increased risk of overall dementia, a 127% increased risk of vascular dementia, and a 56% increased risk of AD associated with diabetes (Gudala et al., 2013). Mid-life vascular risk factors, including diabetes, obesity, high cholesterol, and hypertension, have the most substantial impact on late-onset dementia due to AD (Reitz, 2013).

### 3.2. Dyslipidemia in AD

There is growing evidence of a strong connection between dementia and lipid metabolism, especially in AD, where alterations in lipid levels and distribution play a role in disease progression (Hottman et al., 2014). Multiple studies have linked dyslipidemia to AD, primarily because of elevated levels of total cholesterol and LDL-cholesterol (LDL-C) and reduced levels of HDL-cholesterol (HDL-C) (Launer et al., 2001; Wanamaker et al., 2015). Elevated circulatory HDL-C amounts are associated with a reduced chance of CVD (Miller and Miller, 1975). Furthermore, oxidation-derived stress in the CNS can lead to the oxidation of LDL-C (ox-LDL) and VLDL (ox-VLDL), which determine cytotoxicity of oxidized forms (Escargueil-Blanc et al., 1994). The incidence (Grossi et al., 2018) of the ε4-allele of the APOE gene is higher in AD patients than in controls, which indicates the importance of genetic factors in AD development.

The relationship between cholesterol and AD is well-established, yet the precise mechanisms of cholesterol’s impact on AD pathophysiology remain unclear (Akyol et al., 2021). Brain cholesterol directly affects Aβ-related processing. Reduced cholesterol in hippocampal neurons has been linked to a decrease in Aβ oligomerization (Schneider et al., 2006), whereas elevated cholesterol levels in AD brains promote the activity of γ- and β-secretases (Xiong et al., 2008). In essence, cholesterol may enhance the degradation of β- and γ-secretases in AD brains, leading to elevated Aβ levels. In mild-AD and AD patients, cholesterol ester (CE) levels were higher compared to controls (Akyol et al., 2021). Brain cholesterol levels are primarily regulated by *de novo* synthesis in astrocytes, oligodendrocytes, and neurons (Grimm et al., 2017).

The mechanism connecting AD to the ε4-allele is not yet completely understood. Researchers have proposed a connection between protease activities that degrade Aβ in cerebellar vascular infarction and carriers of the ε4 allele (Zhu et al., 2013). In a study conducted by Koffie et al., AD patients who had the APOE ε4-allele tended to show a significantly greater buildup of Aβ-oligomers than those with the APOE ε3 allele (Koffie et al., 2012). This heightened accumulation of Aβ-oligomers ultimately leads to the loss of synapses.

#### 3.2.1. Lipidomics studies and 27-hydroxycholesterol in AD

A recent study identified specific lipid subclasses associated with more severe AD pathology, including glycerolipids (diacylglycerols [DAG] and triacylglycerols), neutral lipids (CE), glycerophospholipids (phosphatidyl ethanolamine, phosphatidyl glycerol, phosphatidyl inositol, phosphatidylserine, and lysyl-phosphatidylglycerol), sphingolipids, ceramides (CER), dihydroceramides, hexosylceramides, and lactosylceramides (Akyol et al., 2021). Lipidomic studies have shown that AD patients have reduced levels of ether-sterols, PC, sphingomyelin, and phospholipids compared to controls (Oresic et al., 2011). Kim et al. observed a significant increase (more than 2-fold) in 14 lipids in the plasma of individuals with AD, including DAG, phosphatidyl ethanolamine, TG, and CER. Among these lipids PE, DAG, and TG correlated strongly with brain atrophy, making them potential markers for early detection of mild cognitive impairment when combined with MMSE scores (Kim et al., 2013). Trushina et al. used non-targeted metabolomics through liquid chromatography/mass spectrometry and found altered cholesterol and sphingolipid transport in the cerebrospinal fluid and plasma of AD patients compared to cognitively normal individuals (Trushina et al., 2013).

In the periphery, cholesterol is oxidized through the steroid side-chain, resulting in the formation of a compound called 27-hydroxycholesterol (27-OHC). The enzyme CYP27A1, found in almost all cells of the body, is responsible for producing 27-OHC. This mechanism allows cells to mobilize cholesterol as an alternative to the traditional method of HDL-mediated reversed cholesterol transport (Babiker et al., 1997). Recent studies have shown that 27-OHC can cross the BBB and directly enter the cerebral circulation. In a FINGER study, a decrease in 27-OHC levels over a two-year multidomain lifestyle and vascular intervention, which included cardiovascular risk management, dietary modifications, physical activity, and cognitive training, was associated with improved cognitive performance, particularly in the realm of memory (Sandebring-Matton et al., 2021). Furthermore, levels of 27-OHC in the cerebrospinal fluid depend on the integrity and functioning of the BBB. Damage to this barrier increases the transport of 27-OHC from the circulation into the brain (Leoni et al., 2003). The enzyme CYP7B1, found in neurons, is believed to play a critical role in the metabolism of 27-OHC (Meaney et al., 2007). Conversely, the primary mechanism for eliminating cholesterol from the brain involves converting it into a compound called 24S-hydroxycholesterol (24S-OHC), or cerebrosterol, which has the ability to cross the BBB (Bjorkhem et al., 1997). The enzyme CYP46A1, predominantly found in neuronal cells, is responsible for producing 24S-OHC (Lund et al., 1999). There is a reciprocal relationship between these two oxysterols at the BBB, with fluxes occurring in opposite directions. Both fluxes are concentration-driven, with a higher concentration of 24S-OHC in the grey matter, its major site of production, and a lower concentration of 27-OHC in the same area, the main site of metabolism (Heverin et al., 2005). The balance between 24S-OHC and 27-OHC, as well as their metabolites, is hypothesized to be important in amyloidogenesis in AD (Bjorkhem, 2006). Studies have shown a significantly increased ratio of 27-OHC to 24S-OHC in the brains of AD patients, supporting this hypothesis. The flux of 27-OHC into the brain could potentially be the missing connection between hypercholesterolemia and AD.

#### 3.2.2. VLDL and LDL involvement in AD pathology

VLDL receptor polymorphisms have been associated with the risk of dementia, particularly in vascular and mixed dementia (Helbecque et al., 2001). A prospective study revealed an inverse relationship between total VLDL-C and the risk of dementia (Tynkkynen et al., 2018) This may be attributed, at least in part, to the association between cardiovascular events and VLDL-C (Wurtz et al., 2015). Furthermore, one subclass of VLDL lipoproteins (triglyceride-to-total lipid ratio in very-large-VLDL) reduced the risk of dementia, whereas another (total cholesterol-to-total lipid ratio in very-large-VLDL) increased the risk of dementia (Tynkkynen et al., 2018). No significant associations were observed between apolipoproteins, cholesterols, fatty acids, phospholipids, triglycerides, ketone bodies, glycolysis-related metabolites, markers of inflammation, or fluid balance metabolites and dementia/AD (Tynkkynen et al., 2018). In contrast, previous research has indicated that specific phospholipids are linked to the progression of mild cognitive impairment to AD (Mapstone et al., 2014; Li et al., 2016), although the metabolomics platform used in Tynkkynen’s study differs from those in the previous studies.

Linkage analysis, conducted on a genome-wide scale, has identified chromosome-12p as a region of interest for AD, encompassing LDL-receptor-related protein-1, α2-macrogobulin, and lectin-like ox-LDL receptor 1 (OLR-1) as a susceptible region for AD (Wu et al., 1998; Lee et al., 2008). OLR-1, initially discovered in endothelial cells as an ox-LDL receptor (Sawamura et al., 1997), is implicated in the pathogenesis of conditions like diabetes, atherosclerosis, and hypertension (Palmieri et al., 2013). This receptor is fully expressed in the brain and spinal cord (Yamanaka et al., 1998). Inflammatory cytokines, oxidative stress, and ox-LDL can induce its expression (Mehta et al., 2006), and these stimuli may be regarded as a hallmark of AD (Agostinho et al., 2010). OLR-1 functions through ligand interactions, promoting cytokine secretion in brain cells and mitigating the neurotoxic effects of amyloid peptides. Adequate OLR-1 expression may be a key factor in preventing AD. Moreover, it is highly expressed in vascular endothelial cells and may be involved in amyloid angiopathy in the brain, associated with AD progression (Shi et al., 2006). This hypothesis is supported by the research of Lambert et al. (2003), which noted significantly lower OLR-1 mRNA levels in AD patients than in healthy controls.

In healthy individuals, the accumulation of Aβ fibers is effectively prevented by several organized removal mechanisms, including BBB transport, extracellular proteolysis, receptor-mediated endocytosis, and the efflux of soluble Aβ into the peripheral circulation (Ramanathan et al., 2015). The LDL receptor related protein 1 (Lrp-1) plays a pivotal role in Aβ catabolism, internalization, and production (Pietrzik et al., 2002; Deane et al., 2004). Lrp-1 is synthesized on the endothelial cell surface of the BBB, facilitating the rapid removal of Aβ from brain tissue through endocytosis and degradation (Pflanzner et al., 2011). Reduced levels of Lrp-1 in the brains of patients of AD patients have been consistently observed (Shinohara et al., 2017). Aβ forms complexes with lactoferrin, APOE, prion proteins, and activated α2-macrogobulin, which undergo Lrp-1 mediated endocytosis (Kang et al., 2000; Rushworth et al., 2013). The monomeric Aβ also binds to the extracellular ligand-binding domain of Lrp-1, facilitating endocytosis (Deane et al., 2004). Microglial cells predominantly migrate to plaques through micropinocytosis, effectively clearing Aβ (Lee and Landreth, 2010). Lrp-1–mediated Aβ clearance from pericytes helps prevent Aβ accumulation in cerebral vessels (Sagare et al., 2013). Soluble Lrp-1 is cleaved and released from β-secretase in the periphery, further enhancing its binding to Aβ and facilitating its cerebral removal (Sagare et al., 2007). Additionally, Aβ removal from the system is supported by the liver, spleen, and kidney via Lrp-1 (Ramanathan et al., 2015).

#### 3.2.3. Cholesterol metabolism dysfunction in microglia

Emerging evidence suggests that disruptions in cholesterol metabolism, especially in high cholesterol environments, can harm microglia function. When microglia cannot maintain proper cholesterol metabolism and accumulate lipid droplets, it triggers a pro-inflammatory lipid profile (Loving et al., 2021). Excessive cholesterol exposure can negatively affect microglia through membrane protein mechanisms, contributing to various aspects of disease-related microglia dysfunction, including increased inflammation and a reduced ability to engulf particles, both of which are associated with AD. In summary, in high cholesterol settings, elevated cholesterol levels in neuronal cell membranes lead to greater production of Aβ due to the proximity of key proteins involved in Aβ production. This, in turn, burdens microglia with clearing more Aβ. Simultaneously, high cholesterol impairs microglia’s ability to clear Aβ and enhances their inflammatory signaling and production of ROS. These factors drive Aβ accumulation, leading to plaque formation and creating a pro-inflammatory environment that contributes to neurodegeneration. Statin drugs, commonly prescribed and generally safe, may offer a potential approach to regulate cholesterol and reduce neuroinflammation (Munoz Herrera and Zivkovic, 2022). Moreover, a high cholesterol (3%) diet induces a pro-inflammatory response in rodent microglia models by activating the inflammasome. While anti-inflammatory cytokines are also released as part of the response, the ultimate outcome is a compromised BBB (Chen et al., 2018). Moreover, as supported by the literature (Duong et al., 2021), cholesterol overload can lead to chronic inflammation in microglia. This inflammation is further induced by L5, which activates microglia through mechanisms involving an increase in nitrogen/oxygen radicals (peroxides, nitric oxide [NO], and ROS), elevated levels of TNF-α, reduced basal levels of interleukin (IL)-10, and increased production of proinflammatory proteins such as inducible NOS and cyclooxygenase 2 (Yu et al., 2017). L5 predominantly induces apoptosis in BV-2 microglial cells, and this effect is significantly interrupted by signaling pathway inhibitors. This suggests that L5 may interact with toll like receptor-4 to regulate various biopathways, including NF-κB, PI3K/Akt, and MAPKs. These interactions with L5, contributing to atherosclerosis formation, are also implicated in the development of neurodegeneration linked to neuroinflammation (Yu et al., 2017).

#### 3.2.4. Comparing the impact and receptor preferences of L5 and LDL

Circulating LDLs are a group of lipoproteins that have various compositions, electrical charges, and densities. Electropositive LDL and L5 differ in lipid and protein composition, size, and density (Sanchez-Quesada et al., 2012). L5 has much higher aggregation and proteoglycan binding ability, which may be due to its phospholipolytic activity, than does the more positively charged LDL (LDL (+)) (Sanchez-Quesada et al., 2012). L5 promotes inflammatory responses in cells by increasing the expression and secretion of cytokines. L5-releasing inflammatory cytokines were first described in the endothelium (De Castellarnau et al., 2000). By promoting gene transcription, L5 elicits the release of inflammatory mediators like IL-6, IL-10, and monocyte chemoattractant protein-1 (MCP1) (Bancells et al., 2010).

In the PC12 rat cell line, L5 caused cell damage in a time- and concentration-dependent manner (Wang et al., 2017). In a neutral comet assay designed to examine whether L5-induced cell death involved DNA damage, the results showed that L5 and ox-LDL, but not L1 (a less negatively charged form of LDL), led to the formation of comet tails, indicating double-strand breaks in chromatin (Wang et al., 2017). At lower concentrations, L5 hindered nerve growth factor-related neuron cell differentiation by inhibiting the PI3K/Akt cascade through LOX-1-related mechanisms. In the same study, the researchers investigated whether L5 caused apoptotic cell death and activated apoptosis-related pathways in PC12 cells. They found that L5, but not L1, increased caspase-3 expression 6–12 h after exposure and that this apoptotic pathway was blocked by a pancaspase inhibitor called ZVAD-FMK (Wang et al., 2017). Additionally, L5 treatment led to increased levels of phosphorylated-p53 just 10 min after exposure. The study showed that p53-dependent apoptosis is a primary mechanism of cell death induced by L5 in PC12 cells. Furthermore, L5 inhibited differentiation of cultured PC12 cells induced by nerve growth factor, indicating its harmful effects on these cells. The results suggest that LDL(−), particularly the L5 subfraction, is toxic to PC12 cells, reducing their viability in a time- and concentration-dependent manner through LOX-1/p53/caspase-3 dependent apoptosis and ATM/H2AX-related DNA damage. Lowering plasma LDL(−) levels and preventing the electronegative modification of LDL might help prevent cognitive impairment associated with conditions like AD, where high LDL levels are linked to the disease’s progression.

L5 induces cytokine release in monocytes through CD14 toll-like receptor 4 (TLR-4) pathway (Estruch et al., 2013), which is triggered by increased CER content in L5 (Estruch et al., 2014). This signaling pathway has also been associated with the release of proinflammatory cytokine IL-1β (Estruch et al., 2015). Studies have extensively investigated signaling pathways induced by minimally modified LDL and ox-LDL, including activation of PI3k/Akt-mediated p38 MAPK (Rios et al., 2012), ERK (Namgaladze et al., 2008), AP-1 (Choi et al., 2012), and NF-κB transcription factors (Janabi et al., 2000). However, there is limited research on L5-induced signaling pathways, mostly focusing on endothelial cells. In endothelial cells, L5 activates p38 MAPK (Chu et al., 2013; Chen et al., 2015) and transcription factors NF-κB and AP-1 (Chu et al., 2013) while inhibiting the PI3k/Akt pathway (Lu et al., 2009). In monocytes, L5 activated AP-1 and NF-κB (Estruch et al., 2013). Additionally, in one study, L5 facilitated p38 MAPK phosphorylation through the PI3k/Akt1 and TLR4 pathway (Estruch et al., 2016), leading to the activation of NF-κB, CREB, and AP-1, ultimately resulting in cytokine release.

#### 3.2.5. Impact of L5 on endothelial cells and platelets

**Figure 2:**
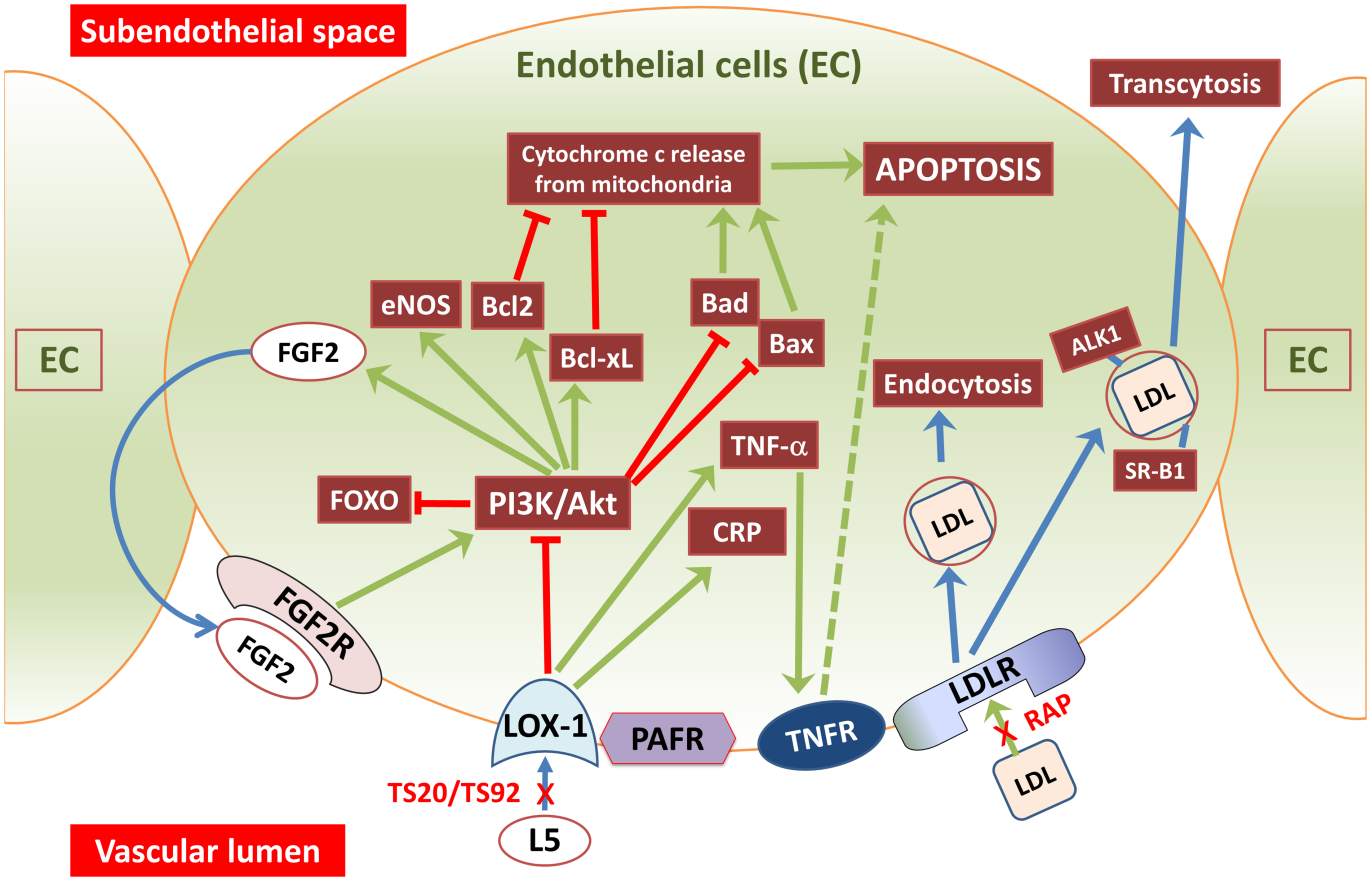
Illustration showing L5 and LDL signaling pathways through vascular endothelial cell membrane receptors. Endothelial cells have receptors for oxidized lipoproteins and machinery for lipoprotein receptor-mediated endocytosis. L5 signals are taken up through LOX-1 and PAFR receptors, whereas regular LDL and its subtypes (L1–L4) are internalized by LDLR. LDLR, a transmembrane glycoprotein found on most cell surfaces, governs LDL uptake and its subsequent lysosomal degradation. LDL is transported through endothelial cells using a process called receptor-mediated transcytosis. This transportation involves receptors like SRB1, ALK1, or LDLR as well as a direct transcytosis mechanism. In contrast, signaling through PAFR and LOX-1 triggers PI3K-PKC activation leading to AKT phosphorylation and reduced cAMP levels, while facilitating granule release. LOX-1 receptors are notably abundant in the human aorta, especially in atherosclerosis-prone areas. Exposure of endothelial cells to L5 causes a three-fold increase in membrane-bound LOX-1 levels. Arrows in the figure indicate the direction or influence of signaling pathways. Green arrows represent stimulation or activation, and red arrows with an end bar signify inhibition or suppression. ALK1, activin receptor-like kinase 1; Akt, protein kinase-B; Bcl2, B-cell lymphoma 2 (antiapoptotic protein); Bcl-xl, B-cell lymphoma extra-large (antiapoptotic protein); CRP, C-reactive protein; EC, endothelial cell; eNOS, endothelial NOS; FGF2, fibroblast-growth factor-2 (basic); FGF2R, fibroblast-growth factor receptor-2; FOXO, forkhead box transcription factor subgroup O; L5, electronegative LDL; LDLR, LDL-receptor; LOX-1, lectin like oxidized LDL receptor 1; PAFR, platelet activating factor-receptor; PI3K, phosphoinositide-3-kinase; RAP, LDL receptor–associated protein; SR-B1, scavenger receptor B1; TNF-α, tumor necrosis factor-alpha; TNFR, TNFα receptor; TS20-TS92, LOX-1 neutralizing-antibodies.

Endothelial cells control the recruitment of blood lymphocytes and monocytes to arterial walls, a crucial step in atherosclerosis. L5 attracts monocytes and lymphocytes to the endothelium (Yang et al., 2003), indicating its involvement in the early stages of atherosclerosis. It also triggers the release of chemokines and adhesion molecules (Figure 2), including vascular-cell adhesion molecules (Ziouzenkova et al., 2003). In human umbilical vein endothelial cells (HUVECs), L5 induced the release of IL-8 and MCP1 (De Castellarnau et al., 2000), which are key factors in recruiting T lymphocytes and monocytes to the endothelium. This effect has been observed in individuals with normal lipid levels (De Castellarnau et al., 2000), familial hypercholesterolemia (FH) (Sanchez-Quesada et al., 2003), and diabetes mellitus (Benitez et al., 2007). In individuals with diabetes and FH, who have higher levels of L5, the inflammatory impact of L5 is likely more pronounced compared to normolipidemic subjects. HUVEC studies have also revealed that L5 triggers the release of various cytokines associated with inflammation, including epithelial cell-derived neutrophil-activating peptide-78 (Abe et al., 2007), granulocyte/monocyte colony stimulating factor, growth-related oncogene, and IL-6 (Benitez et al., 2006). This effect has also been observed in arterial-derived cultured human endothelial cells (de Castellarnau et al., 2007). Lastly, L5 induced the release of matrix metalloproteinases and the expression of vascular-endothelial growth factor in bovine arterial endothelial cells (Tai et al., 2006).

**Figure 3:**
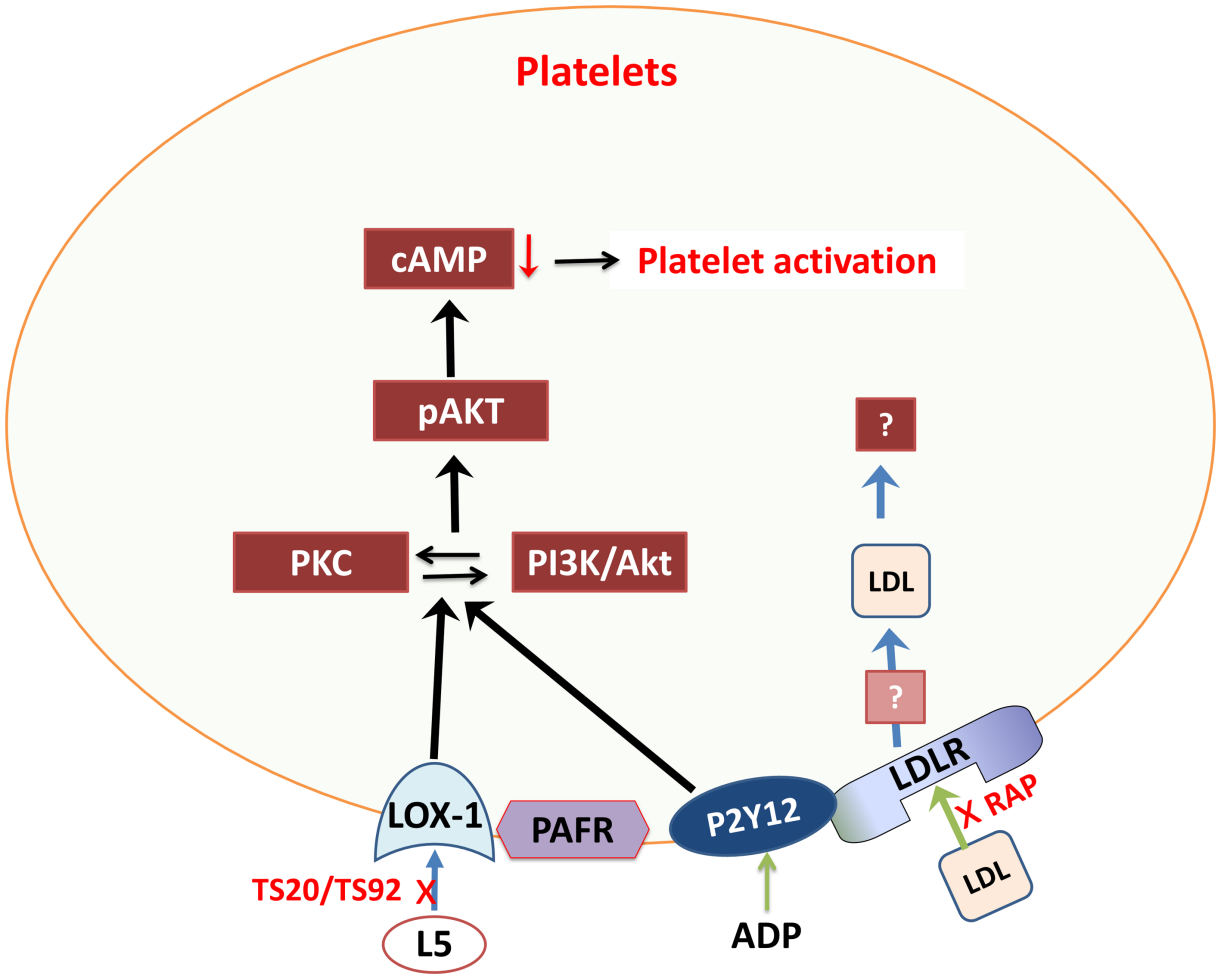
Schematic representation of L5 signaling pathways through various membrane receptors in platelets. Akt, protein kinase-B; cAMP, cyclic AMP; L5, electronegative LDL; LDLR, LDL receptor; LOX-1, lectin-like oxidized LDL receptor 1; PAFR, platelet activating factor receptor; pAKT, phosphorylated protein kinase-B; P2Y12, purinergic receptor P2Y G protein-coupled 12; PI3K, phosphoinositide-3-kinase; RAP, LDL receptor–associated protein; TS20/TS92, LOX-1 neutralizing-antibodies.

Furthermore, research suggests that hypercholesterolemia, particularly with elevated L5 levels, can increase the risk of thrombosis and atherosclerosis (Wang and Tall, 2016; Figure 3). Platelet function and biosynthesis can be affected by hypercholesterolemia, implying that FH patients may not have abnormal platelets but rather may have an issue related to the cholesterol environment where platelets operate. Lowering LDL-C levels can temporarily reduce platelet aggregation. However, it is important to note that this evidence may apply primarily to the specific study participants (Wang and Tall, 2016).

### 3.3. Association between atherosclerosis and AD

Vascular risk factors such as cardiovascular disease (Stampfer, 2006), hypertension (Qiu, 2012), diabetes (Wijesinghe et al., 2016), and CVD (Yarchoan et al., 2012) have emerged as important contributors to AD and have been extensively studied (Rius-Perez et al., 2018). Surprisingly, risk factors related to the vascular system can exacerbate the progression of AD (Li et al., 2010). Vascular issues can lead to reduced blood flow in the brain, hindering the clearance of Aβ and potentially playing a significant role in the development of AD (Gupta and Iadecola, 2015). Atherosclerosis, a condition causing vascular problems, represents a crucial target for reducing the risk of AD risk through intervention and early screening (Lopez-Oloriz et al., 2013).

A growing body of evidence suggests a link between AD and atherosclerosis (Roher et al., 2011). Epidemiologic studies have found common vascular risk factors shared by both conditions, including obesity, smoking, older age, diabetes, hypercholesterolemia, hypertension, APOE4 subtype, and hyperhomocysteinemia (Gorelick et al., 2011). A postmortem study of atherosclerotic occlusion of the circle of Willis arteries revealed that AD patients had more stenosis of major brain vessels than did control subjects (Roher et al., 2011). Atherosclerosis may impede the hyperphosphorylation of tau protein and the accumulation of Aβ by reducing cerebral blood flow (Hansson et al., 2018). Further support for this association comes from a meta-analysis showing a higher prevalence of atherosclerosis in AD patients compared to healthy controls (Xie et al., 2020).

Atherosclerosis can lead to cognitive decline and neuronal damage in several ways. The cerebral hypoperfusion and hypoxia associated with atherosclerosis accelerate the overproduction of Aβ in AD models (Iadecola, 2004). In hypoxic conditions, splitting of Aβ proteins from Aβ precursor protein is markedly increased by over-expression of γ- and β-secretases and downregulation of α-secretase, resulting in increased Aβ production (Salminen et al., 2017). This excess Aβ can harm cerebrovascular function (Iadecola, 2004) and reduce vascular compliance, affecting cerebral blood flow in a pernicious cycle. Atherosclerosis-induced neurovascular disturbance may lead to nitrosative damage and oxidative stress (Chaitanya et al., 2012), which may result in neuroinflammatory responses and metabolic toxicity. Reduced clearance of Aβ coupled with overproduction of Aβ and ROS-induced stress expedites the deposition of Aβ, which eventually leads to cognitive decline (Dore et al., 2013).

### 3.4. Lipid biomarkers in AD

Biomarkers for AD identified through CSF and neuroimaging are primarily confined to scientific research due to high costs, safety concerns, and the need for specialized expertise for clinical application (Sperling et al., 2011). In contrast, blood-based biomarkers, like ApoJ and APOE found in the plasma lipoproteome, offer a cost effective and minimally invasive alternative for early AD detection, making them suitable for widespread clinical use (Sperling et al., 2011). These biomarkers play a crucial role in the progression of AD, making them potentially useful for identifying AD through blood samples. High plasma ApoJ levels and low plasma APOE levels are related to hippocampal atrophy, cerebral amyloidosis, cognitive decline, and dementia, including AD (Song et al., 2012; Gupta et al., 2016). However, it is worth noting that while elevated plasma ApoJ is linked to AD, its diagnostic impact is limited. It improves accuracy only by 8% when combined with factors like APOE genotype, sex, and age (Gupta et al., 2016). Therefore, a methodological approach to enhance the diagnostic value of the plasma lipoproteome should be developed. Fractionating the plasma lipoproteome into distinct classes may reveal unique associations with AD not evident when examining the plasma lipoproteome as a whole. To generate a plasma lipoproteomic workflow, studies involving targeted proteomic methods have been performed based on selected reaction monitoring and liquid chromatography and tandem mass spectrometry as well as relative quantitative peptide analyses (Li et al., 2018).

## 4. Conclusion

AD is a well-recognized neurodegenerative disorder, and its incidence is increasing, positioning it as one of the most impactful diseases alongside cancer and CVD (Brunden et al., 2009). This study provides preliminary evidence to support the innovative idea that quantifying the lipoproteome in the plasma can improve the accuracy of AD diagnosis. Larger scale validation studies are required to test circulating individual lipoproteins in the plasma as potential biomarkers in AD. The current gold standard biomarkers for AD in the CSF, such as tau, phosphorylated tau (p-tau), and Aβ 1–42, require invasive lumbar punctures, making them less suitable for high throughput screening or patient follow-up (Blennow and Hampel, 2003). Thus, exploring blood-based biomarkers, as suggested in our study, holds great promise for improving the accessibility and convenience of AD diagnosis and management in the future.

In this comprehensive review, we have undertaken a detailed exploration of L5. We have shed light on our novel and intriguing hypothesis that L5 may play a pivotal role in initiating neuroinflammation and oxidative stress, which could ultimately contribute to the development of AD. As our knowledge increases and technologies advance, elucidating the pathophysiological mechanisms of AD becomes more feasible in regard to lipoprotein metabolism, particularly the distinctive role of L5. Future research should prioritize the thorough profiling of L5 and the initiation of AD clinical trials designed to unravel the true risk that L5 poses in neurodegeneration. Moreover, our review helps in providing new tools and insights for the development of innovative diagnostics and treatment strategies for AD and other dementia-related diseases. If a strong correlation between L5 and AD pathophysiology is established, the prospect of anti-L5 therapy and antioxidant therapy emerges as a promising avenue for preventing AD symptoms. Anti-L5 therapy, by reducing plasma L5 and inhibiting the electronegative modification of LDL, could emerge as a vital preventive measure against cognitive impairment in AD, even in the absence of a definitive way to halt AD progression. This represents a beacon of hope in the quest to mitigate the devastating impact of AD on individuals and society as a whole.

## Author contributions

OA: Conceptualization, Investigation, Methodology, Visualization, Writing – original draft, Writing – review & editing. SA: Conceptualization, Investigation, Methodology, Visualization, Writing – original draft, Writing – review & editing. M-CC: Conceptualization, Methodology, Writing – review & editing. SC: Conceptualization, Methodology, Writing – review & editing. C-KL: Conceptualization, Methodology, Writing – review & editing. SS: Conceptualization, Methodology, Supervision, Writing – review & editing. JCS: Conceptualization, Methodology, Supervision, Writing – review & editing. C-HC: Funding acquisition, Methodology, Resources, Supervision, Writing – original draft, Writing – review & editing.
